# Do your eye movements reveal your performance on an IQ test? A study linking eye movements and socio-demographic information to fluid intelligence

**DOI:** 10.1371/journal.pone.0264316

**Published:** 2022-03-29

**Authors:** Enkelejda Kasneci, Gjergji Kasneci, Ulrich Trautwein, Tobias Appel, Maike Tibus, Susanne M. Jaeggi, Peter Gerjets

**Affiliations:** 1 Human-Computer Interaction, Department of Computer Science, University of Tübingen, Tübingen, Germany; 2 Data Science and Analytics, Department of Computer Science, University of Tübingen, Tübingen, Germany; 3 Hector Research Institute of Education Sciences and Psychology, University of Tübingen, Tübingen, Germany; 4 School of Education, University of California, Irvine, CA, United States of America; 5 Leibniz-Institut für Wissensmedien, Tübingen, Germany; Sapienza, University of Rome, ITALY

## Abstract

Understanding the main factors contributing to individual differences in fluid intelligence is one of the main challenges of psychology. A vast body of research has evolved from the theoretical framework put forward by Cattell, who developed the Culture-Fair IQ Test (CFT 20-R) to assess fluid intelligence. In this work, we extend and complement the current state of research by analysing the differential and combined relationship between eye-movement patterns and socio-demographic information and the ability of a participant to correctly solve a CFT item. Our work shows that a participant’s eye movements while solving a CFT item contain discriminative information and can be used to predict whether the participant will succeed in solving the test item. Moreover, the information related to eye movements complements the information provided by socio-demographic data when it comes to success prediction. In combination, both types of information yield a significantly higher predictive performance than each information type individually. To better understand the contributions of features related to eye movements and socio-demographic information to predict a participant’s success in solving a CFT item, we employ state-of-the-art explainability techniques and show that, along with socio-demographic variables, eye-movement data. Especially the number of saccades and the mean pupil diameter, significantly increase the discriminating power. The eye-movement features are likely indicative of processing efficiency and invested mental effort. Beyond the specific contribution to research on how eye movements can serve as a means to uncover mechanisms underlying cognitive processes, the findings presented in this work pave the way for further in-depth investigations of factors predicting individual differences in fluid intelligence.

## Introduction

With his theory of human intelligence published in 1963, Cattell [1] established a common understanding of two factors underlying human intelligence: crystallized and fluid intelligence. While crystallized intelligence primarily involves abilities related to acquired knowledge and experience, fluid intelligence encapsulates the general abilities of reasoning and problem solving regardless of such knowledge. As such, fluid intelligence is considered foundational to many cognitive tasks and, most importantly, to learning [2, 3]. Therefore, researchers from various fields have investigated individual differences that contribute to fluid intelligence and—consequently—its derived skills in the areas of learning and cognition. Several approaches have been used to capture individual differences in fluid intelligence, including participants’ reports of strategies [4] or motivational factors (e.g. effort [5, 6]). Given that self-reports can be biased [7], psychophysiological measures, especially ocular movements like scanpaths [8] and pupil diameter [9] have shown to be useful indices for strategies and motivational factors to predict individual differences in fluid intelligence [10]. Thus, to paint a more complete picture of the factors contributing to an individual’s performance in an intelligence test, eye tracking can play an important role as it enables researchers to investigate the participants’ behavior in an unobtrusive way.

### Using eye movements to capture individual differences in fluid intelligence

With the increasing availability of accurate and low-cost eye-tracking technology, new means of monitoring a participant during task accomplishment at a fine-grained level have become available. In particular, there has been an increased interest in employing eye movement analysis to improve understanding of the relationship between eye movements and the allocation of visual and cognitive resources. This interest is reflected in a considerable number of publications, especially in the fields of processing speed and working memory capacity, which are considered key processes underlying performance in fluid intelligence tests [11, 12]. In this context, multiple research articles have also addressed the relationship between eye movements and performance in fluid intelligence measures through empirical studies [8, 10, 13–16].

Prior research has also revealed the pupil to be a particularly interesting eye-related feature, since pupil size changes have been linked to task demands and cognitive effort [17]. In their review, van der Wel and van Steenbergen argue that pupil diameter is an indicator for the general exertion of cognitive effort which tends to be higher for more difficult tasks [17]. Therefore, a more difficult task evokes a greater pupillary response indicating more mental effort and a greater mobilisation of cognitive resources. Already by 1979, Ahern and Beatty demonstrated that the link between task difficulty and pupil diameter in an arithmetic task is moderated by individual differences in intelligence [18]. Individuals with higher intelligence scores responded more accurately and demonstrated a smaller pupil diameter suggesting that individuals with higher IQ scores may need to exert less mental effort to successfully complete the task. In contrast, van der Meer reported an increase in pupil diameter and accuracy in participants with higher fluid intelligence scores solving an analogical reasoning task [19], but only for the most difficult item. Furthermore, Bornemann and colleagues have investigated 11th graders and found a significant positive correlation between task difficulty and pupil dilation for an analogy task that participants were unfamiliar with, but not for an algebra task that was already part of the curriculum [20]. They concluded that the novelty of the task allows participants with greater cognitive abilities to allocate more cognitive resources, whereas a familiar task does not cause this effect. Overall, there seems to be a complex interplay between task difficulty and pupil diameter that varies with task type, novelty, and intelligence.

A limited number of studies have investigated eye-movement patterns and strategies associated with performance in fluid intelligence tests. For example, in a study conducted by Vigneau et al., [10], 55 participants (i.e., university students) were monitored during 14 selected items of the Raven’s Advanced Progressive Matrices test. The authors reported differences in viewing patterns between participants scoring relatively higher or lower on the test. Proportional time on the problem matrix (i.e., test item), latency to first alternation, and time distribution on the problem matrix were positively correlated to test scores, whereas the number of alternations between matrix and response choice and the gaze time spent on answer alternatives were negatively correlated to the test scores [10]. In particular, the authors argue that participants do not only differ with regards to their strategy, but also regarding how that strategy is employed, thereby adding a qualitative dimension to the existing distinction between constructive matching and response elimination [21, 22]. Similar findings were reported by Hayes et al., [14] in a study with 35 university students. The authors found that a significant percentage of the variance on the participants’ scores on a Raven’s Advanced Progressive Matrices test was explained by eye-fixation patterns, where systematic scanning of the problem matrices and less toggling between matrix and responses were indicative of better performance, which likely reflects differential strategies between groups: e.g., more successful participants might have constructed an internal representation of the problem before scanning the answer alternatives. Relatedly, Laurence et al. [23] investigated the association between eye movement patterns and IQ test performance in a study with 34 participants who completed a digitalized version of the Wiener Matrizen-Test 2. The authors reported that participants who scored higher on the test showed less gaze transitions between the relevant areas of interest and the response alternatives [23]. More recently, Sargezeh et al., [13] recorded eye movements of 44 participants while performing a comparative visual search task and found significant differences between participants who scored low, medium, and high on a measure of fluid intelligence using multiple features extracted from eye movements. More specifically, the authors reported a strong positive correlation between saccade peak velocity and test scores, while the ratio of total fixation duration to total saccade duration was negatively correlated with performance [13]. Finally, Curie et al. [24] used a larger sample of 137 participants to investigate the validity of a new visual analogical reasoning paradigm for populations with intellectual disabilities and found that both, problem-solving strategies as well as eye-tracking data explained individual differences in performance. Overall, eye movement patterns seem to be indicative of participants’ strategies, which are associated with successful performance in fluid intelligence tasks.

Although several eye movement features have shown to be useful in predicting individual differences in performance during fluid intelligence tasks, previous research in this domain has often been restricted to relatively small sample sizes. Thus, studies with larger sample sizes are required to reveal robust and reliable results. Our study addresses this research gap and provides a comprehensive analysis of eye-movement features related to task performance using a standardized test of fluid intelligence, the CFT *at the item level*. Furthermore, there is limited research that has focused on participant strategy and their roles in explaining individual differences in problem solving success. Thus, a more comprehensive approach that relies on more fine-grained behavioral and physiological measures and that also includes socio-demographic factors as an additional source of variance is needed to achieve a deeper understanding of the underlying mechanisms contributing to cognitive performance.

### Socio-demographic factors and fluid intelligence

Theoretical accounts from various disciplines including sociology, economics, and psychology as well as empirical evidence suggest a robust link between socio-demographic factors and intelligence. Specifically, socio-economic status that includes the education level of participants and their parents has shown to be significantly correlated with fluid intelligence [25, 26], which has been attributed to differential experiences and opportunities [27, 28]. For example, Kaufman et. al demonstrated the correlation between years of formal education and fluid intelligence in a stratified sample of 1125 adults ranging from 22 to 90 years of age [25]. They found the same correlation for crystallized intelligence and several academic skills. Finally, using a school cut-off design, Zhang et al. [29] found that first-graders outperformed their age-matched kindergarten peers in matrix reasoning, indicating that experiences related to schooling impacted the development of intelligence. Overall, accumulating research has highlighted the importance of educational experiences for cognitive development, which translates to individual differences in intelligence. In addition to educational experiences, other activities have shown to contribute to cognitive performance including various leisure activities such as physical exercise [30], playing computer games [31, 32], and musical training [33]. Collectively, a host of experiential factors have shown to contribute to individual differences in intelligence.

### Research goals

With this work, we aim to advance the literature on individual differences in fluid intelligence by including both, eye-tracking data as well as socio-demographic variables to predict task performance at the item level using machine learning techniques. To address our aims, we rely on the *TüEyeQ* data set [34, 35] collected from 315 university students performing a fluid intelligence test. While a sample size of 315 participants is unusually large in a typical eye movement study, it is rather small-scale compared to other data sets that focus on socio-demographic factors and those in the machine learning literature. While we have hypotheses that are rooted in the body of research on either socio-demography or eye tracking, investigating them jointly has an exploratory character when problem solving success is concerned.

To the best of our knowledge, this is the first study that provides a methodological foundation for the investigation of factors that contribute to individual differences in problem solving skills by relying on socio-demographic, eye movement, and physiological data. More specifically, our predictive model is based on the Gradient Boosting Decision Trees (GBDT) [36] algorithm, which allows us to go beyond conventional linear statistical methods that are restricted to simple relationships between the features and the target variable. The GBDT algorithm is an ensemble approach that makes use of simple decision trees as base learners. Since each tree added to the ensemble is different from the previous trees and focuses on the remaining error, the GBDT algorithm helps to reduce bias [37, 38], which is very important for data sets of moderate size where the instance-related bias and the variability across instances can negatively influence a predictive model. A further advantage of the GBDT algorithm is that it is not vulnerable in the presence of collinearity and is, therefore, very well suited for processing behavioural and eye-tracking data in a holistic way.

## Materials and methods

The TüEyeQ data set was recently published on Nature Scientific Data to enable researchers to freely access the experimental data [35]. Therefore, for a thorough description of the experimental setup and further details on the data, we refer the reader to the data set description as published in [35]. In the following, we will briefly describe the data collection and processing steps of TüEyeQ relevant to this work.

More specifically, let *P* be the set of participants and *I* the set of items of a CFT 20-R fluid intelligence test. For a feature-based description **x**_*P*_(*p*) of a participant *p* ∈ *P* and a feature-based description **x**_*I*_(*i*) of a CFT 20-R item *i* ∈ *I*, we aim to predict whether *p* will correctly solve *i*, that is, we search for a mapping *f*: *concat*(**x**_*P*_(*p*), **x**_*I*_(*i*)) ↦ {0, 1} that can correctly predict whether a participant *p* will succeed or fail on an item *i*, where *concat*(**x**_*P*_(*p*), **x**_*I*_(*i*)) denotes the concatenation of the feature vector describing the participant and the feature vector describing the item. Note that **x**_*P*_(*p*) can vary depending on whether we use only socio-demographic information to describe the participant, or only eye-movement data, or both.

For a detailed description of the features contained in the TüEyeQ data set, we refer to Table 1 and [35].

**Table 1:** Description and encoding of all performance-related, educational and socio-demographic features in the order of their appearance in the csv file as provided by the TüEyeQ data set (available through the Harvard Dataverse Repository under https://doi.org/10.7910/DVN/JGOCKI).

Variable Nr.	Feature	Description	Encoding
1	TaskID	Unique identifier for every task	String, CFT-block-related task id
2	subject	Unique identifier for every participant	String-based id
3	age	The age of a participant	categorical
4	gender	The gender of a participant, i.e. male, female, unknown	categorical
5	handedness	Indicates whether the participant is right-handed or left-handed	binary
6	native_german	This variable describes whether a participant is a native German	binary
7	native_german_mother	Indicates whether the mother of the participant is a native German	binary
8	native_language_mother	The native language of the participant’s mother	categorical
9	native_german_father	Indicates whether the father of the participant is a native German	binary
10	native_language_father	The native language participant’s father	categorical
11	education_mother	The scholarly or professional education of the participant’s mother	categorical
12	education_father	The scholarly or professional education of the participant’s father	categorical
13	training_mother	The scholarly or professional training of the participant’s mother	categorical
14	training_father	The scholarly or professional training of the participant’s father	categorical
15	books	Indicates how many books are in the participant household	categorical
16	job_mother	The profession of the participant’s mother	categorical
17	job_father	The profession of the participant’s father	categorical
18	year_of_degree	The year in which the final study degree was achieved by the participant	categorical
19	mean_grade_degree	The average grade of the participant’s final degree	continuous
20	programming_experience	Indicates whether the participant has experience programming languages	binary
21	smartphone_usage	Indicates the frequency of smartphone usage (range: never to daily)	categorical
22	tablet_usage	Indicates the frequency of tablet usage (range: never to daily)	categorical
23	notebook_usage	Indicates the frequency of notebook usage (range: never to daily)	categorical
24	desktop_pc_usage	Indicates the frequency of desktop pc usage (range: never to daily)	categorical
25	tv_usage	Indicates the frequency of tv usage (range: never to daily)	categorical
26	text_editor_usage	Indicates the frequency of text editors usage (range: never to daily)	categorical
27	spreadsheet_usage	Indicates the frequency of spreadsheet software usage (range: never to daily)	categorical
28	presentation_software_usage	Indicates the frequency of presentation software usage (range: never to daily)	categorical
29	email_usage	Indicates the frequency of email usage (range: never to daily)	categorical
30	browser_usage	Indicates the frequency of web browser usage (range: never to daily)	categorical
31	google_usage	Indicates the frequency of Google usage (range: never to daily)	categorical
32	wikipedia_usage	Indicates the frequency of Wikipedia usage (range: never to daily)	categorical
33	facebook_usage	Indicates the frequency of Facebook usage (range: never to daily)	categorical
34	twitter_usage	Indicates the frequency of Twitter usage (range: never to daily)	categorical
35	skype_usage	Indicates the frequency of Skype usage (range: never to daily)	categorical
36	youtube_usage	Indicates the frequency of Youtube usage (range: never to daily)	categorical
37	ebay_usage	Indicates the frequency of Eabay usage (range: never to daily)	categorical
38	amazon_usage	Indicates the frequency of Amazon usage (range: never to daily)	categorical
39	online_news_usage	Indicates the frequency of online news usage (range: never to daily)	categorical
40	online_banking_usage	Indicates the frequency of online banking usage (range: never to daily)	categorical
41	gaming_adventure	Indicates whether the participant primarily plays adventure games	binary
42	gaming_action	Indicates whether the participant primarily plays action games	binary
43	gaming_first_person_shooter	Indicates whether the participant primarily plays first person shooter games	binary
44	gaming_casual	Indicates whether the participant primarily plays casual games	binary
45	gaming_mmo	Indicates whether the participant primarily plays Massive Multiplayer Online games	binary
46	gaming_racing	Indicates whether the participant primarily plays racing games	binary
47	gaming_rpg	Indicates whether the participant primarily plays Role Playing Games games	binary
48	gaming_simulation	Indicates whether the participant primarily plays simulation games	binary
49	gaming_sports	Indicates whether the participant primarily plays sports games	binary
50	gaming_strategy	Indicates whether the participant primarily plays strategy games	binary
51	smoking	Indicates whether the participant is a smoker	binary
52	excessive_drinking	Indicates whether the participant is an excessive drinker	binary
53	grades_math	The participant’s final math grade (German Abitur)	continuous
54	grades_german	The participant’s final German grade (German Abitur)	continuous
55	grades_biology	The participant’s final biology grade (German Abitur)	continuous
56	grades_physics	The participant’s final physics grade (German Abitur)	continuous
57	grades_chemistry	The participant’s final chemistry grade (German Abitur)	continuous
58	grades_geography	The participant’s final geography grade (German Abitur)	continuous
59	grades_history	The participant’s final history grade (German Abitur)	continuous
60	grades_art	The participant’s final art grade (German Abitur)	continuous
61	gaming_hours_weekly_min	The minimum hours the participant spends gaming per week	continuous
62	gaming_hours_weekly_max	The maximum hours the participant spends gaming per week	continuous
63	leisure_simple_entertainment	Indicates whether the participant’s leisure activity involves simple entertainment	binary
64	leisure_mental_activity	Indicates whether the participant’s leisure activity involves mental activity	binary
65	leisure_sports_exercise	Indicates whether the participant’s leisure activity involves sports and exercise	binary
66	leisure_music	Indicates whether the participant’s leisure activity involves music	binary
67	leisure_art	Indicates whether the participant’s leisure activity involves art	binary
68	leisure_dance	Indicates whether the participant’s leisure activity involves dance	binary
69	leisure_hobbies	Indicates whether the participant’s leisure activity involves hobbies (e.g. DIY)	binary
70	leisure_play_games	Indicates whether the participant’s leisure activity involves playing (video-) games	binary
71	leisure_relaxation	Indicates whether the participant’s leisure activity involves relaxation	binary
72	leisure_social_activity	Indicates whether the participant’s leisure activity involves social activities	binary
73	leisure_humanitarian_services	Indicates whether the participant’s leisure activity involves humanitarian work	binary
74	leisure_nature_activities	Indicates whether the participant’s leisure activity involves nature/outdoor activities	binary
75	leisure_travel_tourism	Indicates whether the participant’s leisure activity involves travel and tourism	binary
76	study_subject_primary	The primary study subject category of the participant	categorical
77	study_subject_secondary	The secondary study subject category of the participant	categorical
78	cft_sum_full	The aggregated CFT score of the participant	continuous
79	cft_task	Indicates whether the participant solved the task correctly	binary
80	fixationCount	The number of fixations performed by a participant during a test item	continuous
81	meanFixationDuration	The mean duration of fixations performed by a participant during a test item	continuous
82	saccadeCount	The number of saccades performed by a participant during a test item	continuous
83	meanSaccadeAmplitude	The mean amplitude of saccades performed by a participant during a test item	continuous
84	meanSaccadeDuration	The mean duration of saccades performed by a participant during a test item	continuous
85	microsaccadeCount	The number of microsaccades that occured during a test item	continuous
86	meanMicrosaccadeAmplitude	The mean amplitude of microsaccades during a test item	continuous
87	meanMicrosaccadeDuration	The mean duration of microsaccades during a test item	continuous
88	meanMicrosaccadePeakVelocity	The mean peak velocity of microsaccades during a test item	continuous
89	meanPupilDiameter	The mean pupil diameter of a participant during a test item	continuous

### The TüEyeQ data set

#### Study participants

TüEyeQ contains data collected from 315 healthy participants (217, female, 94 male, 4 not stated; with an age mean of 23.272 years, SD 3.022) completing the CFT-R, and providing their socio-demographic and educational background characteristics, including information on leisure time activities and the use of technology, software, and gaming (see Table 1 for a complete list of variables). Due to technical shortcomings or low tracking quality, eye movement data is available for only 229 out of these 315 participants as will be explicitly described in the following. All participants had a university entrance qualification, reported no neurological or psychiatric pre-existing conditions, and no visual impairment above 3 dioptres. The Ethics Committee at the Psychological Institute at the University of Tübingen confirmed that the procedures were in line with ethical standards of research with human subjects. All participants were informed in written form and consented that their anonymous data can be analyzed and published. Due to a self-constructed pseudonym, they had the option to revoke this consent at any time.

#### The IQ test

The participants performed the first part of the revised version of the “culture fair” intelligence test (CFT 20-R) designed by Weiß et al. [39]. This test is intended to measure the general mental capacity (i.e., the g-factor of intelligence or fluid intelligence) by means of different problem types that require the ability to recognize figural relationships and to engage in formal logical thinking in problems of varying degrees of complexity under a time restriction. The CFT 20-R consists of four categories of different problem types, namely series continuation, classifications, matrices, and topological conclusions. Each of these categories consists of 11–15 test items with increasing difficulty and a time limit of 3–4 minutes.

In order to record the eye movements of the participants during the task, we adapted the classic pen-and-paper version of the IQ test to a digital one that can be displayed on a computer screen. To imitate paper version as closely as possible, we presented several test items on a single screen page as long as this did not necessitate scrolling. Further information regarding the presentation and layout of the test can be found in [35].

#### Data acquisition

As described in [35], the data was collected in a digital classroom equipped with 30 remote eye trackers attached to laptops with 17inch HD display screens running at full brightness. This setup allows for data collection of up to 30 participants simultaneously, minimizing the overall time needed for collection. For this study, verbal instructions were given to the entire group pertaining to a brief overview of the protocol and an explanation of eye tracking, then individual calibrations were performed with a supervised quality check. Interactions between the participants and the computer took place via mouse or touch pad depending on participants’ preference.

The collection environment controlled the room illumination level, ensuring no effects from sunlight or other outdoor light. The standard maintained illuminance for the experimental sessions was between 10 to 50 lux, measured with a Lux sensor (i.e., Gossen Mavo-Max illuminance sensor, MC Technologies, Hannover, Germany).

The study participants first received general instructions about the nature of the test, followed by the first block consisting of one particular type of problem. Each block had specific instructions, introducing the participants to the block’s requirements and demonstrating the essence of the problem types based on three exemplary test items. The instruction phase was conducted without time constraints, thus all participants could go through the examples and familiarize themselves with the test procedure. All instructions were presented in German using the SoSci Survey online platform [40].

#### Eye-tracking equipment

Eye movement data were collected by means of SMI RED250 remote eye trackers, a commercial eye tracker with 250Hz sampling frequency. Since the eye tracker has a high sampling frequency, both stable (fixations) and rapid (saccadic) eye movements for static stimuli can be measured. Eye movements were recorded using the included eye-tracking software Experiment Center which outputs the raw gaze data consisting of x and y coordinates of each data point, the timestamp information, and the pupil diameter in millimeters.

Calibration was performed for all participants. A validation also was performed as a quality check to measure the gaze deviation for both eyes from a calibration point. A deviation larger than one degree required re-calibration. Calibrations were performed prior to the experiments as well as one or two times during the experimental session depending on how many images were presented.

#### Quality of eye tracking data

Initially, the raw gaze data was examined for signal quality using the eye-tracking software BeGaze provided along with the eye trackers. This software reports the proportion of valid gaze signal to stimulus time as the tracking ratio. Therefore, if a participant’s tracking ratio was deemed insufficient (i.e., less than 80% for at least a part of the task), we omitted the corresponding eye-tracking data. This pre-processing stage can assure that errors (e.g. post-calibration shifts, poor signal due to glasses) in the gaze data are substantially minimized. Consequently, eye-tracking data from 58 participants had to be omitted due to low tracking ratios. An additional 11 eye-tracking data sets were excluded due to errors in the presentation software and another 17 because of incomplete data. This leaves us with eye-tracking data for only 229 of 315 participants. The raw eye-tracking data was then pre-processed to improve the data quality and to extract the relevant features.

#### Eye movement features

Building on previous work that has focused on the investigation of individual differences in fluid intelligence using eye-movement data, our aim was to uncover the extent to which specific eye-movement features can predict CFT performance at the item level. More specifically, the selection of eye-movement features used in our work is informed by previous work; that is, we focus on such features that have shown to be indicative of specific strategies during problem solving as discussed in the introduction. Beyond the typically used features, e.g., fixation or saccade related features, we also include some additional features (e.g. microsaccades, pupillometry) that are more exploratory. Following eye-movement features as provided by the TüEyeQ data set were considered in our models:

- Fixation-related information—Fixations describe the periods where the eye is “still” and thus perceiving visual information. Fixations were extracted from the raw eye-tracking data based on the I-VT algorithm [41]. In our models, we use both the number of fixations, i.e., fixationCount, as well as their mean duration, i.e., meanFixationDuration, during an IQ-test item, since previous work relates longer fixations to higher processing load and more effort [42].
- Microsaccades-related information—Microsaccades, i.e., fixational eye movements, occur during an especially prolonged fixation, and have been previously linked to visual attention [43, 44], perception [45], working memory [46], and task difficulty [47]. In this work, we used the following features related to microsaccades from the TüEyeQ data set: microsaccadeCount (i.e., the mean number of microsaccades that occurred during a particular CFT task performance), meanMicrosaccadeAmplitude (i.e., the mean amplitude of microsaccades that occurred during each item of the CFT), meanMicrosaccadeDuration (i.e., the mean duration of microsaccades that occurred during problem solving), meanMicrosaccadePeakVelocity, (i.e., the mean peak velocity of microsaccades during problem solving).
- Saccade-related information—The TüEyeQ data set also provided saccade-related information. Saccades are rapid eye movements that enable us to change the focus of attention and were extracted from the eye-tracking protocols based on the I-VT algorithm [41] with a velocity threshold of 30°/*s*. Since saccade velocity depends on neural activity and cannot be voluntarily controlled [48], saccade parameters have been previously linked to fluid intelligence. In our analysis, we employ the following parameters of saccades from TüEyeD: saccadeCount (corresponding to the number of saccades occurring during problem solving), the meanSaccadeAmplitude ((i.e., the mean amplitude of saccades occurring during problem solving), and the meanSaccadeDuration (i.e., the mean duration of saccades accruing during problem solving).
- Pupillary information—Since pupil diameter has been used used as an indicator of cognitive load, short-term memory, language processing, reasoning, perception, sustained attention, and selective attention [49–56], we also include the mean pupil diameter in our data analysis (meanPupilDiameter) and explicitly investigate its role in determining problem solving success.

### Feature and factor analysis

**Fig 1:**
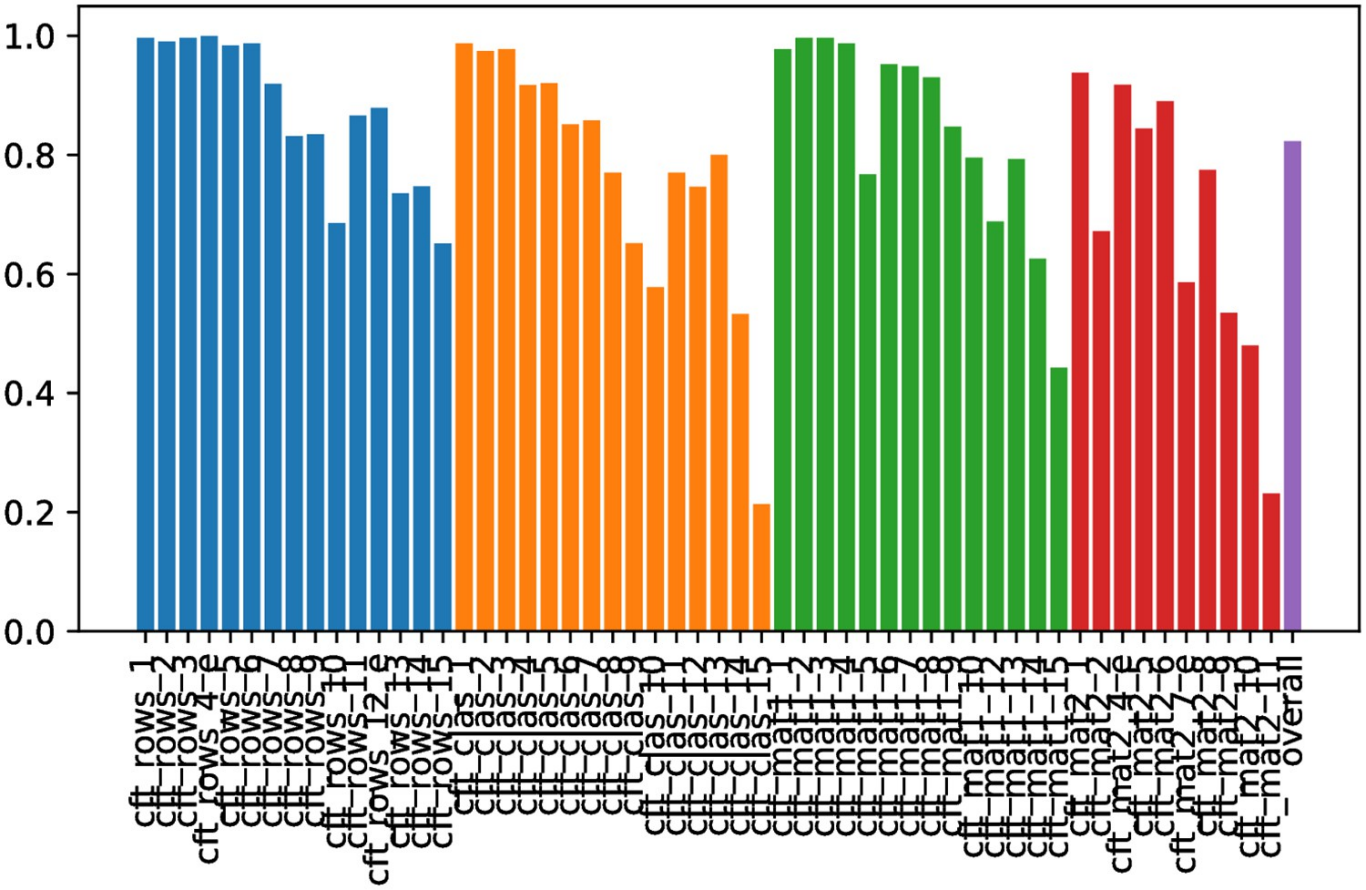
Success rates for each item of the CFT and a summary regarding all responses combined. The purple bar (on the very right) stands for the average success rate across all test items. The remaining colors encode the four different blocks of the test.

Table 1 provides a detailed description of all features provided by the TüEyeQ data set. In addition to that, Fig 1 shows the percentage of correct responses for each test item and the average success rate (i.e., across all items in purple).

To analyse the socio-demographic and eye-movement features with respect to their variability, we first removed the columns related to the following features from the data set: participant (i.e., the id of a participant), taskID (i.e., the id of an item in the CFT 20-R. Note that the feature taskID encodes both the CFT 20-R category/block to which the task belongs and the position of the task within that category), cft_task (i.e., a binary variable indicating whether the CFT item was solved correctly), and cft_sum_full (i.e., the total score of a participant over the CFT 20-R). After removing these features, we were left with a total of 85 features, 10 of which are eye-movement features.

In a next step, we developed a predictive model on the TüEyeQ data set with the goal of reliably predicting the performance of a participant on a random CFT item as described by its taskID, which gives hints at both the item type (i.e., one of the four task blocks of the CFT 20-R framework) and the position of the item within the corresponding CFT 20-R task block (i.e., of items of the same type). An increasing index of taskID (i.e., as represented by the ending digits) corresponds to increasing task difficulty.

In this work, we also conduct a feature importance analysis on the prediction of a participant’s CFT task performance and find that some features are, indeed, highly discriminative from a statistical point of view.

### Predicting task performance: Model description

To identify an adequate predictive model for the TüEyeQ data, we conducted an empirical evaluation of various machine learning algorithms on the data. Not surprisingly, a predictive model based on the Gradient Boosting Decision Trees (GBDT) algorithm [36] showed the highest predictive performance. Our empirical findings on the excellent predictive performance of GBDT are also supported and complemented by previous results from numerous Data Science competitions and challenges. More specifically, according to [57],among the 29 winning solutions of Kaggle challenges (https://www.kaggle.com/competitions) in 2015, 17 solutions used the GBDT algorithm.

The GBDT algorithm is an ensemble approach that makes use of simple decision trees as base learners. A new decision tree *t*_*k*_ is added at step *k* to optimize $L^{k}=\sum_{j=1}^{n}{\left(y_{j}-\left(t_{k}\left(x_{j}\right)+{\hat{y}}_{j}^{k-1}\right)\right)}^{2}+\sum_{i=1}^{k}\Omega \left(t_{i}\right)$, where *n* is the number of training instances, *y*_*j*_ is the true label of **x**_*j*_, ${\hat{y}}_{j}^{k-1}$ denotes the prediction for **x**_*j*_ based on the *k* − 1 decision trees used so far, and *t*_*k*_ represents the new decision tree. Ω(⋅) is a regularisation term, which imposes constraints on the tree structures. The above loss can be minimized through stochastic gradient descent hence the name of the approach. Interestingly, the above loss function can be reformulated in a way that yields a clear strategy for the growing procedure of the current tree (i.e., whether or not to continue splitting a node, which feature to use, etc.).

Since each tree added to the ensemble is different from the previous trees and focuses on the remaining error, the GBDT algorithm helps to reduce bias [37, 38], which is very important for data sets of moderate size where the instance-related bias and the variability across instances can negatively influence a predictive model. Another advantage of the GBDT algorithm is that it is not vulnerable in the presence of collinearity and is, therefore, very well suited for processing behavioural and eye-tracking data. Moreover, the GBDT algorithm has high application value since it can deal effectively with missing values and does not require much data preprocessing (apart from turning the target variable into a nominal variable). Note that this is a strong advantage over other advanced ML algorithms (e.g. Deep Learning) because most of the information contained in the data can be maintained, which is highly beneficial to the prediction quality.

For the development of our GBDT-based model, we used the LGMBClassifier from the LightGBM framework (https://lightgbm.readthedocs.i0). Our model employed 100 decision trees with a maximum depth of 7 for each tree, a learning rate of 0.1, and a scale_pos_weight parameter that compensates for the class imbalance in the dataset. All other parameters were left in their default configuration. The script that was used in our analysis is provided as a supplementary file to ensure transparency and reproducibility of our results.

#### Validation

To provide robust estimations and exploit the training data as effectively as possible, we adopted a stratified group 20-fold cross-validation strategy. Thus, the largest part of the data (i.e., over 16.000 instances) was used for training and other 800 examples for testing. It also ensured that data from any participant is only ever present in either the training or the validation set. Note that although there is some variance in the predictive performance across the 20 folds, as can be seen in the Fig 3a–3c, the standard deviation of the ROC curves is within an acceptable range.

#### ROC-AUC measure

The ROC-AUC (i.e., the area under the receiver operating characteristic curve) quantifies the performance of a classification model over all classification score thresholds. The ROC curve plots two parameters: (1) the True Positive Rate, i.e., $tpr=\frac{TP}{TP+FN}$, and (2) the False Positive Rate, i.e., $fpr=\frac{FP}{FP+TN}$. Note that the *tpr* is a synonym for the recall of a predictive algorithm whereas the *fpr* represents the rate of false alarms. The ROC curve plots the *tpr* vs. the *fpr* values at different classification score thresholds. It can be shown that the area under the ROC curve is the ranking accuracy with respect to the classification score returned by a classifier. Ideally, instances that belong to the positive class should be assigned a higher score by the classifier and thus ranked higher than the instances that belong to the negative class. Hence, an AUC of 1 means that all positive instances are ranked before the negative instances and the two classes are clearly separated by the classifier. In contrast, an AUC of 0.5 means that there is no order across the instances and, as a result, the classes cannot be separated.

### Explainability approach

In Machine Learning, post-hoc explanability techniques can help gain insight into the importance of input features for predictions made by a complex model $f:R^{n}\to \left[0,1\right]$ (e.g., an ensemble model that uses *n* different input features like the GBDT model described earlier). Two of the most popular techniques, so-called local attribution frameworks, are described in [58–60]. The main idea behind local attribution explainability is to generate a local attribution score for each feature by optimizing a simple (typically linear) explanation model *g* such that it locally approximates the complex model *f*. Hence, *g* can be seen as a local interpolation of *f* in the region of interest, i.e., in the close neighborhood of an input $x\in R^{n}$, where *n* is the number of input features.

One of the most widely used local attribution techniques that comes with a strong semantic interpretation of a feature’s importance was introduced in [58, 59]. It approximates the Shapley value [61] of a feature to quantify it’s local attribution score. The Shapley value originates from Cooperative Game Theory and is a value that represents a player’s contribution to the result achieved by a coalition of players. In terms of predictive modelling, the Shapley value determines the marginal contribution of an input feature to the prediction for all possible combinations of inputs. Specifically, according to the original formalisation in [61], given a feature vector $x={\left(x_{j}\right)}_{j=1}^{n}$, let $\phi_{j}\in R$ be the Shapley value of input feature $x_{j}\in R$:

$$\begin{array}{c} \phi_{j}=\sum_{S\subseteq F\backslash \{j\}}\frac{|S|!(|F|-|S|-1)!}{|F|!}[f_{S\cup \{j\}}\left(x_{S\cup \{j\}}\right)-f_{S}\left(x_{S}\right)], \end{array}$$ (1)

where *F* denotes the original set of features and |⋅| denotes the cardinality of a set. Besides, *f*_*S*_(*x*_*S*_) is the prediction of model *f* based on the input features that are included in the subset *S*. For practical purposes, the other features (i.e., in *F*\*S*) are not removed; instead they are set to baseline values [62]. We can reformulate (1) in terms of a characteristic function *v*(*S*) to express *ϕ*_*j*_ as the expectation of the marginal contribution of feature *x*_*j*_:

$$\begin{array}{c} \phi_{j}=\sum_{S\subseteq F\backslash \{j\}}v\left(S\cup \left\{j\right\}\right)-v\left(S\right)=\sum_{S\subseteq F\backslash \{j\}}\Delta_{j}v\left(S\right)=E_{S}\left[\Delta_{j}v\left(S\right)\right] \end{array}$$ (2)

For the computation of the Shapley value, we would have to consider 2^|*F*|^ feature subsets, which is not feasible for high-dimensional data. Hence, various approximations of (1) have been proposed [58, 63, 64]. For the explainability analysis in this work, we employ the TreeExplainer approach presented in [58].

## Results

In this section, we first report the results of the statistical tests on the eye-movement features. More specifically, we investigated the differences between participants either solving the item correctly (“task solved”, representing one item within the CFT) or answering the item incorrectly (“task not solved”) using all eye-movement features provided by the TüEyeQ data set. We further examined the predictive information as drawn from the socio-demographic features, eye-movement features, as well as from the combination of all features, respectively. Finally, we investigate the differential impact of the implemented features on the prediction made by the machine learning model.

### Eye-movement data

In a first step, we conducted statistical tests with regard to the eye-movement information in the TüEyeQ data set. More specifically, a t-test to compare items that were solved correctly and those that were answered incorrectly. The results of this statistical comparison are shown in Table 2.

**Table 2:** Statistical comparison of the eye-movement features during items that were solved correctly vs. those that were answered incorrectly.

Eye-movement feature	Cohen’s d correct vs. incorrect	p-value correct vs. incorrect	Incorrectly answered		Correctly solved	
			Mean	SD	Mean	SD
fixationCount	-0.65	≤ 10^−161^	29.07	21.71	17.16	13.89
meanFixationDuration [ms]	-0.13	≤ 10^−5^	582.07	243.81	549.95	262.63
saccadeCount	-0.66	≤ 10^−163^	29.76	22.20	17.20	13.99
meanSaccadeAmplitude [px]	0.10	≤ 10^−4^	223.70	75.58	230.94	75.52
meanSaccadeDuration [ms]	0.04	0.12	28.08	4.97	28.28	4.80
microsaccadeCount	-0.02	0.51	1.91	1.31	1.89	1.39
meanMicrosaccadeAmplitude [px]	-0.02	0.53	7.47	7.14	7.34	7.66
meanMicrosaccadeDuration [ms]	-0.02	0.51	11.48	7.85	11.33	8.36
meanMicrosaccadePeakVelocity [px/frame]	-0.02	0.43	23.48	18.76	23.04	19.43
meanPupilDiameter [mm]	0.14	≤ 10^−6^	3.38	0.33	3.43	0.33

**Fig 2:**
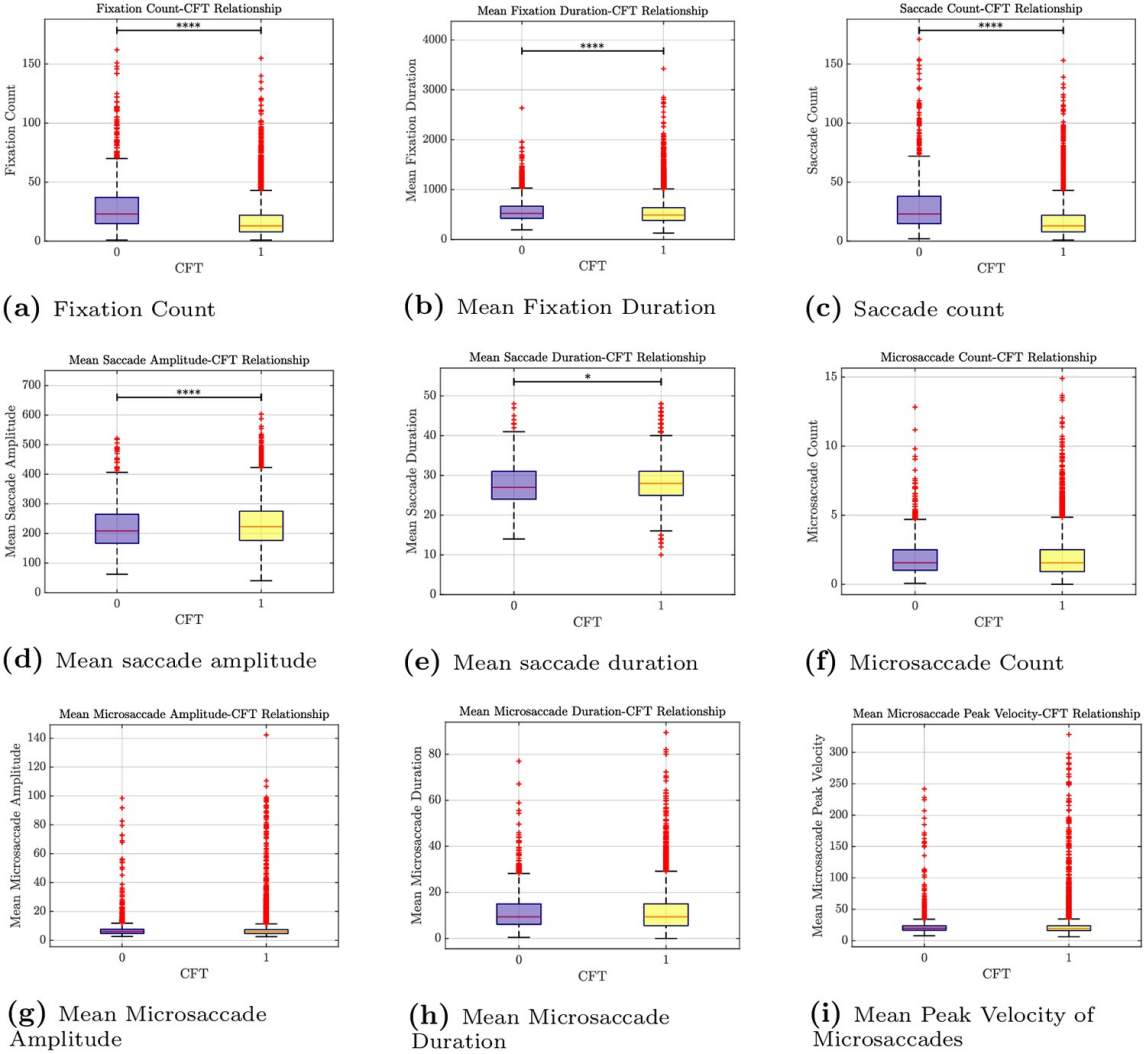
Eye movement differences between the incorrectly answered (purple) and correctly solved items (yellow). (a) Fixation Count. (b) Mean Fixation Duration. (c) Saccade count. (d) Mean saccade amplitude. (e) Mean saccade duration (f). Microsaccade Count. (g) Mean Microsaccade Amplitude. (h) Mean Microsaccade Duration. (i) Mean Peak Velocity of Microsaccades.

As presented in Table 2 and shown in Fig 2a, our results show a highly significant difference (*p* < 0.0001) between fixation counts on items that were answered incorrectly (29.07±21.71) and and those that were solved correctly (17.16±13.89). More specifically, CFT items that were solved correctly were characterized by significantly less fixations than CFT items that were incorrectly answered by the participants. Our results show similar findings with regard to the mean fixation duration (Fig 2b), indicating significantly shorter fixations for correctly solved CFT items (549.95ms±262.63ms) than for items that were answered incorrectly (582.07ms ± 243.81).

Consistent with the previously described data, we found highly significant differences regarding the saccade-related features SaccadeCount (i.e., the number of saccades) and meanSaccadeAmplitude. As shown in Table 2 and Fig 2c, in the case of correctly solved CFT items, the participants performed significantly less saccades than for test items that were incorrectly answered. Furthermore, during CFT items that were solved correctly, participants performed saccades with significantly larger amplitudes than during items that were incorrectly answered, see also Fig 2d. With regard to the feature MeanSaccadeDuration, we found no significant difference between the two conditions. As shown in Table 2 and in the Fig 2f–2i, there were no significant differences for miccrosaccade-related parameters between the two behaviours (task-solved vs. task not solved).

With regard to the pupil diameter size, we found a highly significant difference between the the items that were solved correctly and those which were not. As shown in Table 2, participants showed a larger pupil diameter size in the case of correctly solved test items as compared to those items that were answered incorrectly.

### Predictive information in the features

Our goal was to evaluate the impact of the features related to eye-movements on predicting whether a participant successfully solved a given CFT item. To this end, we built three GBDT models with the same number of decision trees, i.e., 100, the same maximum depth of 7 per tree, and the same learning rate of 0.1. All three GBDT models were trained and validated by applying the cross-validation procedure as introduced above in the model description.

The first GBDT model was trained on the eye-movement-related features only. The second GBDT model was trained only on the socio-demographic features, and the third was trained on the combined 85 features. There are two questions of interest:

- Are the features related to eye movements informative enough for predicting the performance of a participant on a given CFT item?
- If so, is the information contained in the eye-movement features complementary to the information contained in the socio-demographic features? Or, more specifically, is a predictive model developed on both types of features, i.e., the eye-movement and the socio-demographic features, more discriminative than the models developed on the single subgroups of features?

**Fig 3:**
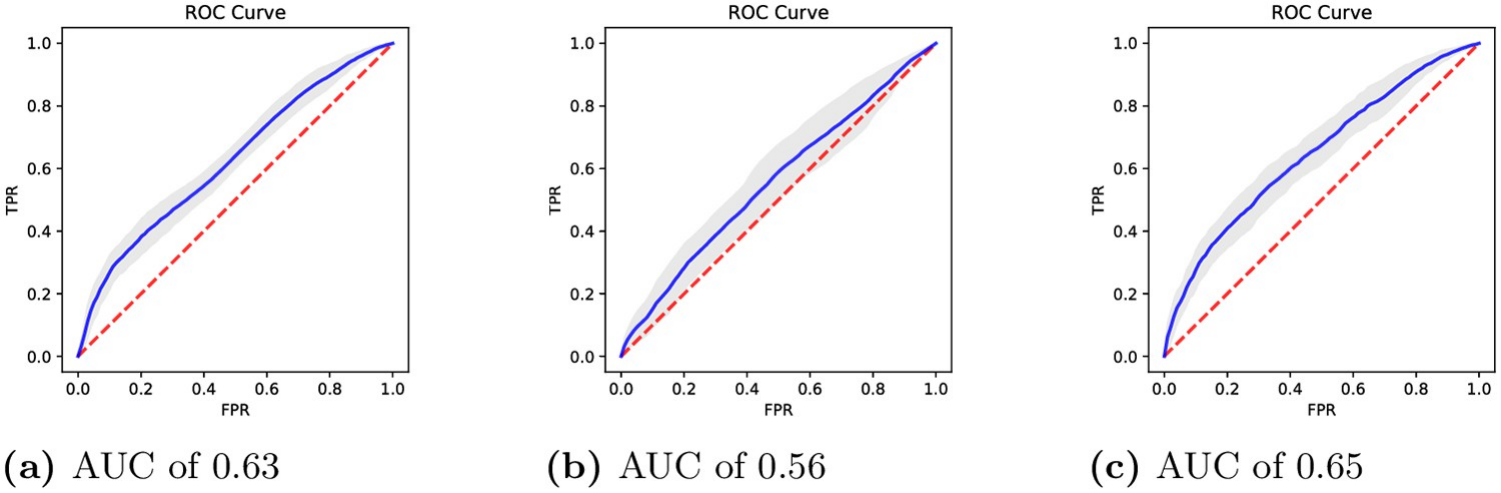
ROC curves of the GBDT model on the TüEyeQ data set that was trained on (a) only the features related to eye-movements, (b) only the socio-demographic features, and (c) on the socio-demographic and eye-movement-related features. Standard deviations based on the 20 folds of cross-validation are shown in gray. (a) AUC of 0.63. (b) AUC of 0.56. (c) AUC of 0.65.

Interestingly, as it can be seen in Fig 3a, the GBDT model developed on the eye-movement features alone is already discriminative with an ROC-AUC of 0.63. Note that the model uses only 10 features (i.e., the features related to eye-movements in the TüEyeQ data set). The GBDT model developed on the 75 socio-demographic features is, as shown in Fig 3b, less discriminative, with an ROC-AUC of 0.56. However, as depicted in Fig 3c, the socio-demographic and the eye-movement-related features contain complementary information, and thus, the GBDT model developed on all features is the most discriminative, with an ROC-AUC of 0.65. Note that this difference with regard to the discriminative performance of the model is substantial, and thus, highlighting the complementary contribution of eye movement features and socio-demographic features to the predictive model.

### Explainability results

**Fig 4:**
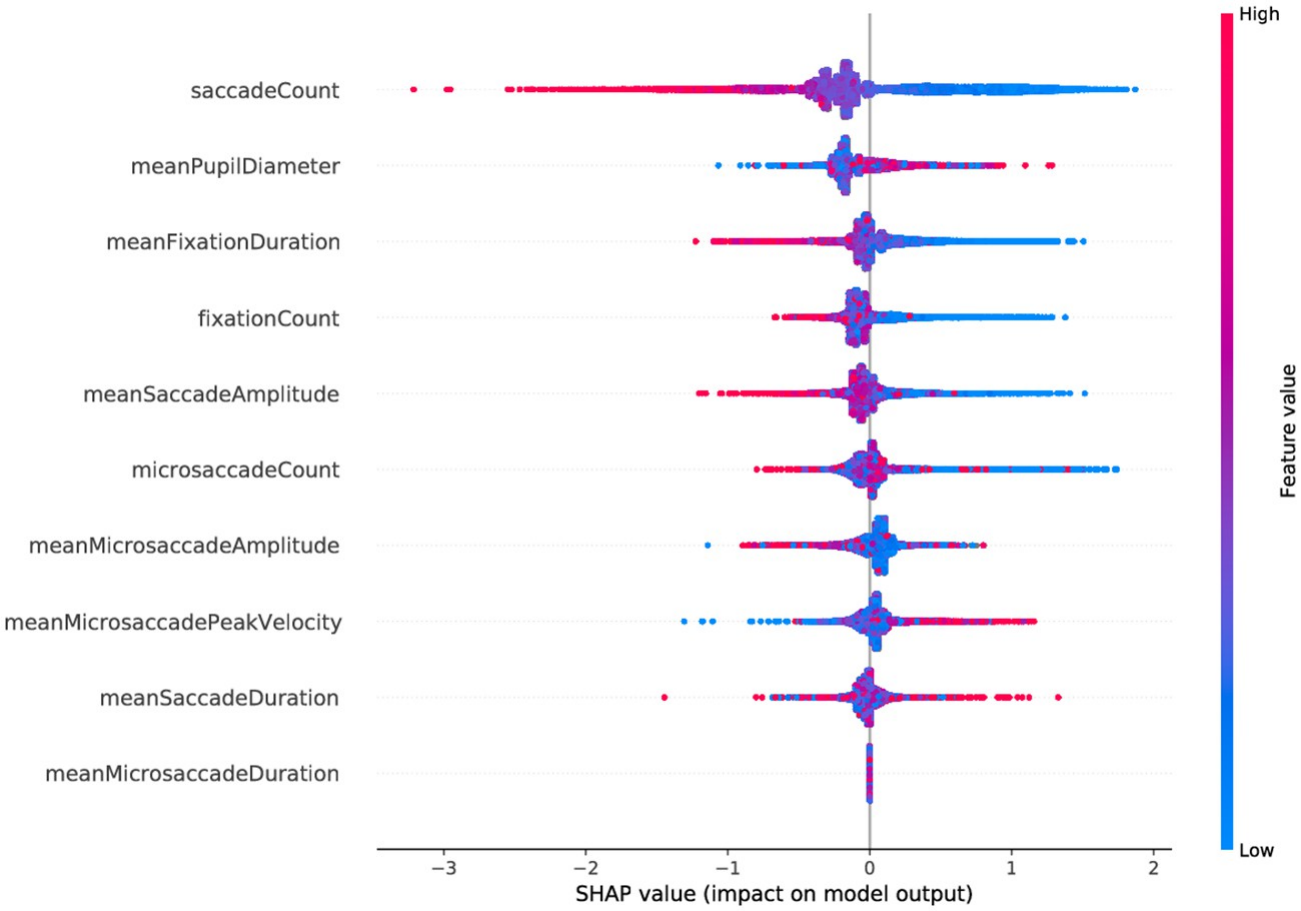
Summary plot of the approx. Shapeley values, that is, the density of the marginal contributions of the features in the GBDT model that uses only the eye-movement features. Red denotes high feature values, whereas blue indicates low feature values, and grey show categorical values or missing values that cannot be assigned a feature value.

Fig 4 shows that the features saccadeCount and meanPupilDiameter followed by meanFixationDuration and fixationCount provide the maximum information according to their marginal contribution density for the GBDT model that uses only the eye-movement features. It also becomes apparent that less saccades, a lower mean fixation duration and a larger mean pupil diameter contribute to the success of solving a item of the CFT.

**Fig 5:**
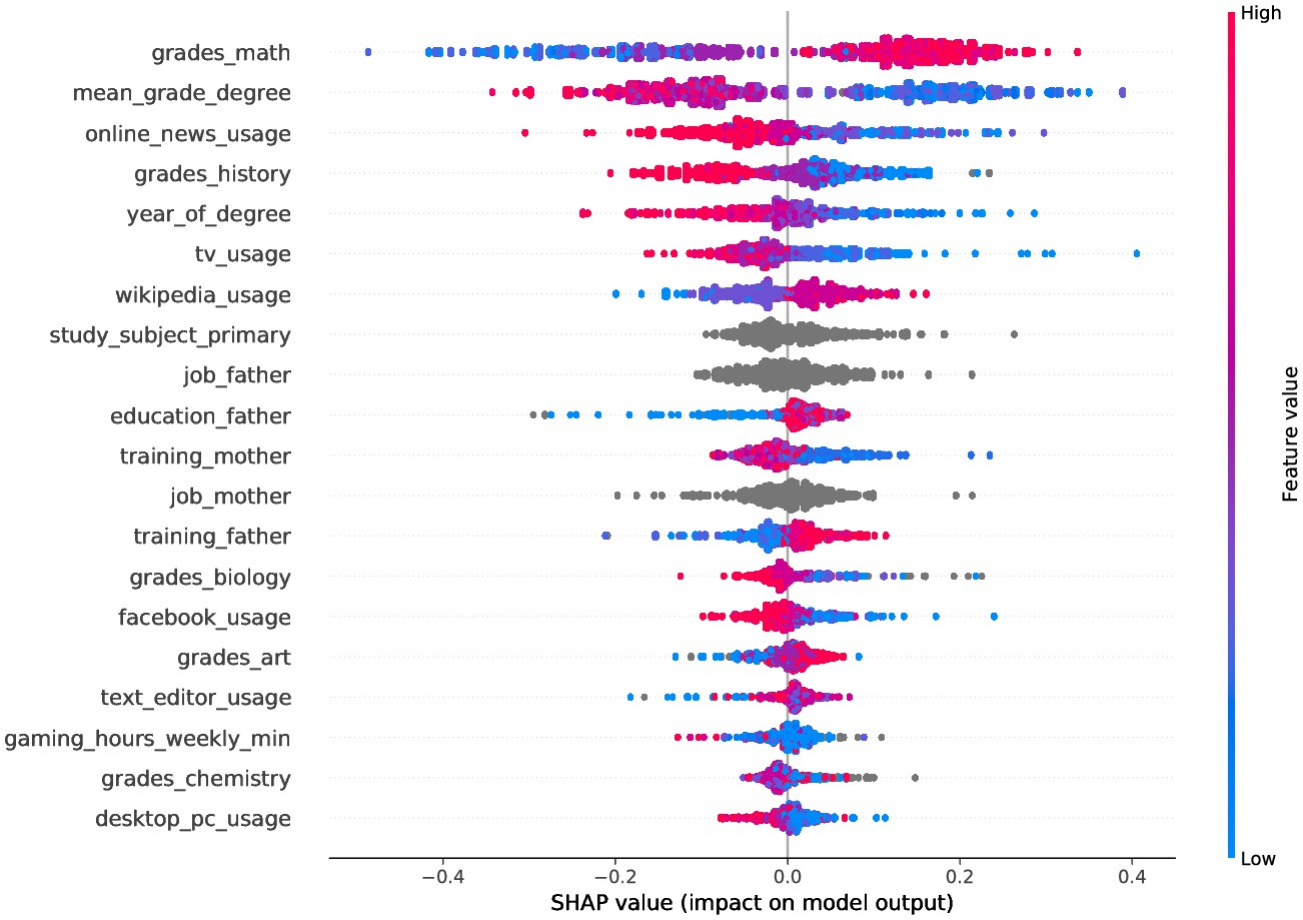
Summary plot of the approx. Shapeley values, that is, the density of the marginal contributions of the features in the GBDT model that uses only the socio-demographic features. Red denotes high feature values, whereas blue indicates low feature values, and grey show categorical values or missing values that cannot be assigned a feature value.

Fig 5 shows that the features grades_math, mean_grade_degree, online_news_usage followed by background information on digital affinity and parental education/jobs provide the maximum information according to their marginal contribution density for the GBDT model that uses only socio-demographic features. Good grades in mathematics and participants’ current standing in their study subject seem to indicate better performance in the CFT, while most forms of media consumption imply the contrary.

**Fig 6:**
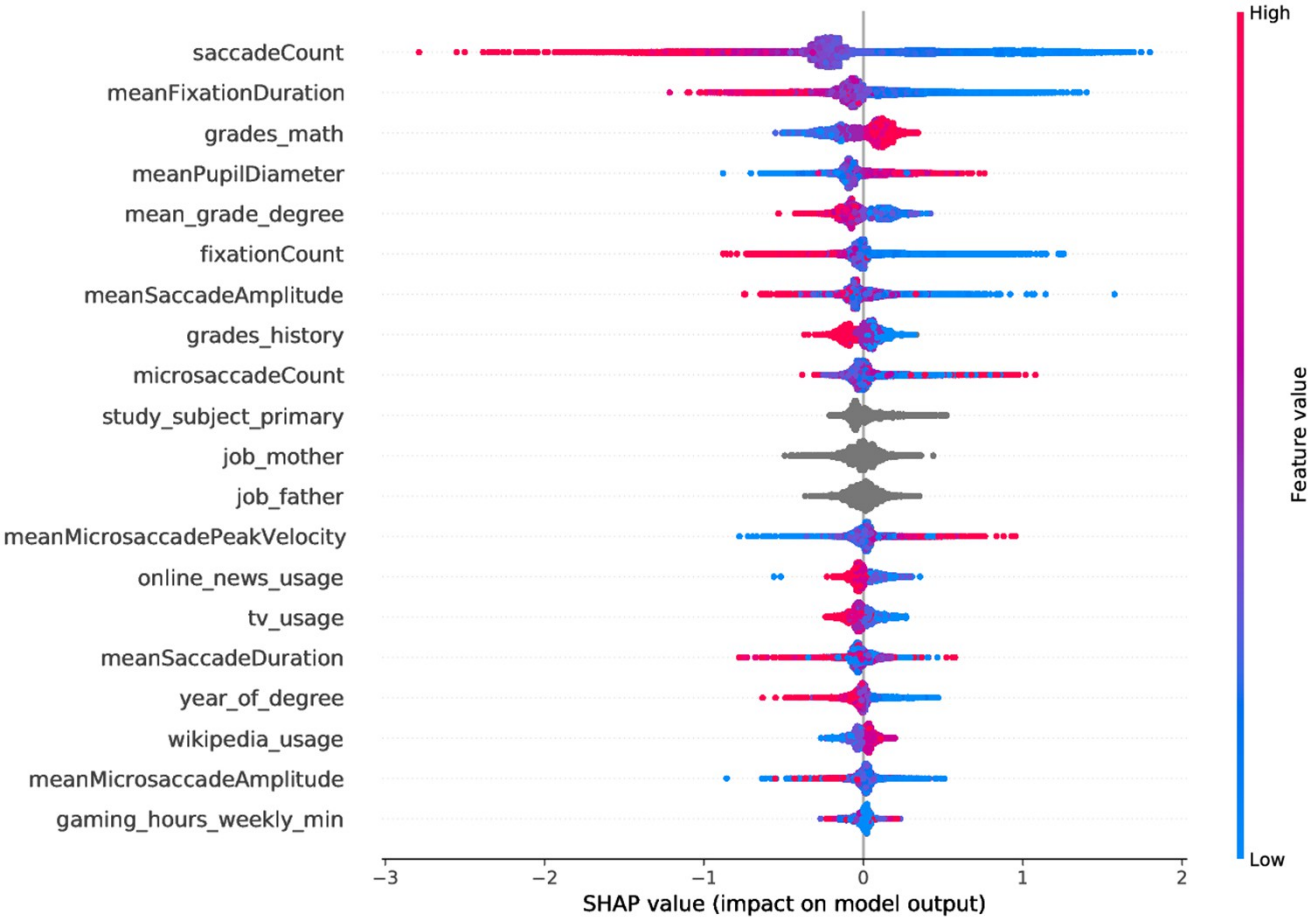
Summary plot of the approx. Shapeley values, that is, the density of the marginal contributions of the features in the GBDT model that uses only eye related and socio-demographic features. Red denotes high feature values, whereas blue indicates low feature values, and grey show categorical values or missing values that cannot be assigned a feature value.

In Fig 6 we can see that five of the seven most informative features are eye-movement related—with grades_math and mean_grade_degree being the exceptions. The feature importances regarding eye movements are very similar to the ones already shown in Fig 4 and again indicate that saccade count, pupil diameter, and fixation duration carry information about the success while solving individual test items. Additionally, participants’ primary subject of study and their parents occupation play an important role as highlighted by their distributions. This is also in line with the importances that were presented in Fig 5.

## Discussion

Following the structure of the result sections, we will first discuss our findings from the statistical tests on the eye-movement features, followed by a discussion on the predictive power of eye movements, socio-demographic information, as well as the overall combined feature set, respectively.

### Statistics of the eye-movement features

#### Fixation-related features

As presented in Table 2 and Fig 2a, both fixations-related features (i.e., fixation count and mean fixation duration) showed that there was a highly significant difference between those items that were solved correctly and those which were not. During CFT items that were solved correctly, participants showed fewer fixations as compared with test items that were answered incorrectly, indicating faster processing speed and higher confidence. Additionally, correctly solved CFT items were characterized by shorter fixation durations, indicating more confidence in extracting and processing (visual) information. Our results are well in line with previous work that has reported associations between fixation duration and memory and processing load [13, 65, 66]. Furthermore, they support the general literature that has emphasized the relationship between fixation properties and performance in executive function and fluid intelligence tests, e.g., [13, 67, 68].

#### Saccade-related features

As reported in Table 2, participants performed significantly less saccades for test items that were correctly solved than for those CFT items that they answered incorrectly. Additionally, in the case of correctly solved items, the participants performed saccades with significantly larger amplitude and longer duration. These results are in line with findings from related work [23], indicating an efficient visual search strategy, and/or successful retrieval of mental representations [69]. Along this line, Sargezeh et al., [13] also reported a strong positive correlation between saccade peak velocity and performance in fluid intelligence tasks [13].

#### Microsaccade-related features

With regard to the microsaccade-related features (i.e., the mean number of microsaccades, the mean microsaccade amplitude and duration, and the mean peak velocity of microsaccades) we found no significant differences between the CFT items solved correctly and those that were answered incorrectly. Although the relationship between microsaccades, working memory [46, 70], and task difficulty [47] is gaining increasing research interest, our results did not show significant differences between the items solved vs. items not solved with regard to these features, despite the fact that the items that were answered incorrectly were generally more difficult. There might be different explanations for these results. First, the eye-tracking data provided by the TüEyeQ data set was captured at 250Hz, which might be too low to thoroughly study microsaccade-related features. In addition, current literature reports inconsistent findings with regard to the underlying nature of microsaccades. While the majority of published papers consider this type of eye movements involuntary, others show that microsaccades can easily be triggered externally, e.g., [71, 72], and thus raising the question of how to interpret microsaccades.

#### Pupil diameter

As shown in Table 2, we found a significant difference in the mean pupil diameter during problem solving as a function of items that were correctly solved vs. those answered incorrectly. Chen and Epps [73] report a smaller pupil response for tasks that overload participants as compared to tasks that participants successfully perform with very high load. The smaller pupil diameter that we observed for items that were answered incorrectly might reflect this cognitive overload.

### A machine learning perspective on the data

We trained three GBDT models (i.e., using the eye-movement features, the socio-demographic information, and all 85 features) from the TüEyeQ data set to investigate the following questions:

Q1 Are the features related to eye-movements informative enough for predicting the success of a participant in solving a given CFT item?
Q2 If the previous question can be positively confirmed, is the information contained in the eye-movement features complementary to the information contained in the socio-demographic features? Or, more specifically, is a predictive model developed on both types of features, i.e., the eye-movement and the socio-demographic features, more discriminative than the models developed on the single subgroups of features?

The results from the GBDT model trained on only ten eye-movement features show that information contained in the eye movements is very discriminative (ROC-AUC of 0.63 as shown in Fig 3a). Our findings regarding Q1 are well in line with related literature, showing, thus, a significant association between eye-movement properties and fluid intelligence [13]. Going beyond previous research which investigated a subset of these features in a rather fragmented way based on small sample sizes, our findings are based on a considerably larger sample size and sophisticated machine learning algorithms. With regard to Q2, we found socio-demographic information as captured by the GBDT model developed on the remaining 75 socio-demographic features to be less predictive (Fig 3b, ROC-AUC of 0.56) than the GBDT model on the eye-movement features. The machine learning model reveals even better predictive performance once all socio-demographic and eye-movement-related features are included, which confirms our assumption that information contained in these feature subsets is complementary and can be combined to significantly improve the predictive performance on whether a CFT item will be solved correctly by an individual.

Our explainability model confirmed that the eye-movement features with the most significant differences between both groups (task solved correctly vs. task not solved), i.e., saccadeCount, meanPupilDiameter, meanFixationDuration, and fixation count, have the highest impact for classification. Interestingly, multiple features derived from the microsaccades were revealed to significantly impact the model’s prediction. However, we did not find any significant differences between the microsaccade-related features with regard to the items that were solved vs. those that were not solved, which might be also related to the fact that the sampling rate of our eye-tracking devices (250 Hz) does not allow to capture fine-grained information on microsaccades. Fig 4 shows that the features microsaccadeCount and meanMicrosaccadeAmplitude have a high impact for classification to the positive class (i.e., item solved correctly). In contrast, meanMicrosaccadePeakVelocity has a high impact for classification to the negative class. Further research is needed to investigate the manifestation of fluid intelligence on microsaccade-related features and their causality.

The results of our explainability analysis regarding socio-demographic factors are in line with previous research in the literature [74–76]. Our analyses show a positive association between parental education level and occupation and an individual’s performance on the CFT. Furthermore, students’ academic background was also associated with performance on the CFT, confirming the findings in existing literature as well [25].

Importantly, our results show that a model that combines eye-movement and socio-demographic features performs significantly better than models trained exclusively on either of the two, suggesting that these two sources of information are complementary. This is supported by the explainability analysis that found 9 eye-related features and 11 socio-demographic features to be the 20 most predictive features for success in a given item of the CFT. Additionally, features that performed well in the combined model were also predictive in their respective single-category model, further backing the conclusion that eye movements and socio-demographic information contribute differential variance to the model.

### Limitations and future work

Although the overall number of participants in our study is higher than that of related studies, it is important to note that all participants were university students, and as such, it is unclear whether the results will generalize to other populations. In our future work, we, therefore, aim to further investigate the contribution of individual differences on problem solving success in a more diverse population using eye-movement patterns [77, 78]. Here, we focused primarily on general eye-related features and investigated their predictive power on solving individual CFT items. As revealed by our explainability model, multiple features derived from the microsaccades significantly influenced the prediction of the machine learning model. Since the sampling rate of our eye-tracking devices was only 250 Hz, these results can only be considered indications and require further investigations to gain insights on the relationship between microsaccades and problem solving success. Finally, there are additional sources of variance to consider that were not included here (but that could be added to the model), and thus, this work is a first step into using this approach to explain variance in problem solving success using a broad range of variables. Going one step further, counterfactual explanations [79, 80] could not only help identify important features/factors for predicting a person’s performance on a problem, but also help develop individual strategies for efficient problem solving.

## Conclusion

We found that specific eye-movement patterns are related to the ability of a participant to succeed in solving a given CFT item. Moreover, the eye-movement information is complementary to the socio-demographic information in predicting individual differences in problem solving success within the context of a standardized fluid intelligence test, suggesting that each source of information contributes important (but distinct) variance. Our method of analysis is based on a computational framework with machine learning and explainability at its core and thus, goes beyond purely correlational results. The sample size that we employed is considerably larger than what is typical in related research, which allowed the utilization of an extensive and rich feature set, while still maintaining the validity of our results, as demonstrated by our use of cross-validation. Overall, our computational framework that relies on a machine learning and explainability approach, might facilitate and thus contribute more in-depth investigations of a broad set of factors predicting individual differences in higher cognitive functions using large populations.

## Supporting information

S1 File (PY)
